# Apoptosis-Related Gene Expression Profiling in Hematopoietic Cell Fractions of MDS Patients

**DOI:** 10.1371/journal.pone.0165582

**Published:** 2016-11-30

**Authors:** Saskia MC Langemeijer, Niccolo Mariani, Ruth Knops, Christian Gilissen, Rob Woestenenk, Theo de Witte, Gerwin Huls, Bert A van der Reijden, Joop H Jansen

**Affiliations:** 1 Department of Laboratory Medicine, Laboratory of Hematology, Radboud University Medical Center (Radboudumc), Nijmegen, The Netherlands; 2 Department of Hematology, Radboud University Nijmegen Medical Centre, Nijmegen, The Netherlands; 3 Department of Human Genetics, Radboud University Medical Center, Nijmegen, The Netherlands; 4 Department of Tumor Immunology, Radboud University Nijmegen Medical Centre, Nijmegen, The Netherlands; 5 Department of Hematology, University Medical Center Groningen, Groningen, The Netherlands; St. Vincent's Institute, AUSTRALIA

## Abstract

Although the vast majority of patients with a myelodysplastic syndrome (MDS) suffer from cytopenias, the bone marrow is usually normocellular or hypercellular. Apoptosis of hematopoietic cells in the bone marrow has been implicated in this phenomenon. However, in MDS it remains only partially elucidated which genes are involved in this process and which hematopoietic cells are mainly affected. We employed sensitive real-time PCR technology to study 93 apoptosis-related genes and gene families in sorted immature CD34+ and the differentiating erythroid (CD71+) and monomyeloid (CD13/33+) bone marrow cells. Unsupervised cluster analysis of the expression signature readily distinguished the different cellular bone marrow fractions (CD34+, CD71+ and CD13/33+) from each other, but did not discriminate patients from healthy controls. When individual genes were regarded, several were found to be differentially expressed between patients and controls. Particularly, strong over-expression of *BIK* (*BCL2-interacting killer*) was observed in erythroid progenitor cells of low- and high-risk MDS patients (both p = 0.001) and *TNFRSF4* (tumor necrosis factor receptor superfamily 4) was down-regulated in immature hematopoietic cells (p = 0.0023) of low-risk MDS patients compared to healthy bone marrow.

## Introduction

Myelodysplastic syndromes (MDS) represent a heterogeneous group of malignant hematopoietic disorders that are characterized by dysplasia in the myeloid, megakaryocytic and/or erythroid cell lineages. The clinical course of MDS is highly variable. Whereas some patients suffer from smoldering cytopenias, others rapidly develop a more aggressive disease eventually resulting in acute myeloid leukemia (AML). The WHO classification and International Prognostic Scoring systems (IPSS and IPSS-R) recognize the heterogeneity of MDS and divide patients into subgroups based on characteristics such as the amount of blasts in the bone marrow and the number of cytopenias [1–4].

Although cytopenias are present in the peripheral blood of the vast majority of MDS patients, the bone marrow is usually normocellular or hypercellular. Apoptosis of hematopoietic cells in the bone marrow has been implicated in this phenomenon. Several studies have shown signs of increased apoptosis in bone marrow of MDS patients, using techniques such as *in situ end labeling* (ISEL) of fragmented DNA/ TUNEL assay [5–7], electron microscopy [8, 9], flowcytometry using annexin V staining [10–12] and measurement of mitochondrial membrane potential [11]. The percentage of cells affected by apoptosis differs between studies, possibly due to the use of different techniques and the heterogeneity of the clinical samples studied. In most, but not all studies apoptosis markers are particularly elevated in the more indolent cases of MDS, whereas apoptosis is reduced or at normal levels in the more aggressive cases. This led to the hypothesis that apoptosis is initially increased in MDS due to either primary defects of the apoptotic pathway or in response to oncogenic stress. During the advanced stages of MDS, this response may be lost as a result of further transformation of the malignant cells. The presence of apoptosis has been studied in whole bone marrow and in several bone marrow fractions, such as CD34+ cells. Depending on the study, increased apoptosis parameters were found in the CD34+ cell fraction of all MDS patients [11, 12] or only in patients with the more indolent phenotype [10]. Others described that apoptosis is mainly increased in the more committed myeloid, erythroid and/or megakaryocytic lineages [8, 12, 13].

Apoptosis is a tightly regulated process that involves many proteins. The differential regulation of some of these proteins, such as Fas [12, 14], FLIP [13, 15], BCL-2 and BCL-2-related proteins [10, 16–21], TNF proteins and their receptors [22–25], IAPs [7, 26–28] and caspases [19, 29–32] has been the subject of studies in MDS patients. Most of these studies focused on only one or a few apoptosis-related genes and were performed in either whole bone marrow or the CD34+ cell fraction. Based on the results of these previous studies, we hypothesized that a distinct apoptotic gene expression signature might be present in specific hematopoietic stem and progenitor fractions from healthy individuals compared to MDS patients. To gain insight into the expression patterns of apoptosis-related genes, we have employed sensitive real-time PCR technology to study 93 apoptosis-related genes and gene families in both CD34+ immature hematopoietic cells as well as differentiated erythroid (CD71+) and monomyeloid (CD13/33+) cells.

## Results

### Isolation of hematopoietic cell fractions

Hematopoietic cell fractions were isolated from the bone marrow of 23 patients and 10 healthy controls (S1 Table). Three different hematopoietic cell fractions were sorted, CD34+ cells (immature hematopoietic cells), CD71+ erythroid precursors and CD13/33+ mono-myeloid precursors. According to the IPSS score 4 low, 11 int-1, 4 int-2 and 4 high risk patients were included. In most cases we were able to isolate all three subfractions from the same patient (S1 Table). As a control, the expression of several genes related to erythroid differentiation (*TFRC*, *GYPA*, *GYPB*, *GYPC*, *SPTB*, *SPTA1*, *EBP41*, *EBP42* and *HBB)* was measured in all fractions and shown to be highly upregulated in CD71+ cells (data not shown).

### Unsupervised cluster analysis separates different bone marrow fractions but does not differentiate MDS patients and controls

**Fig 1:**
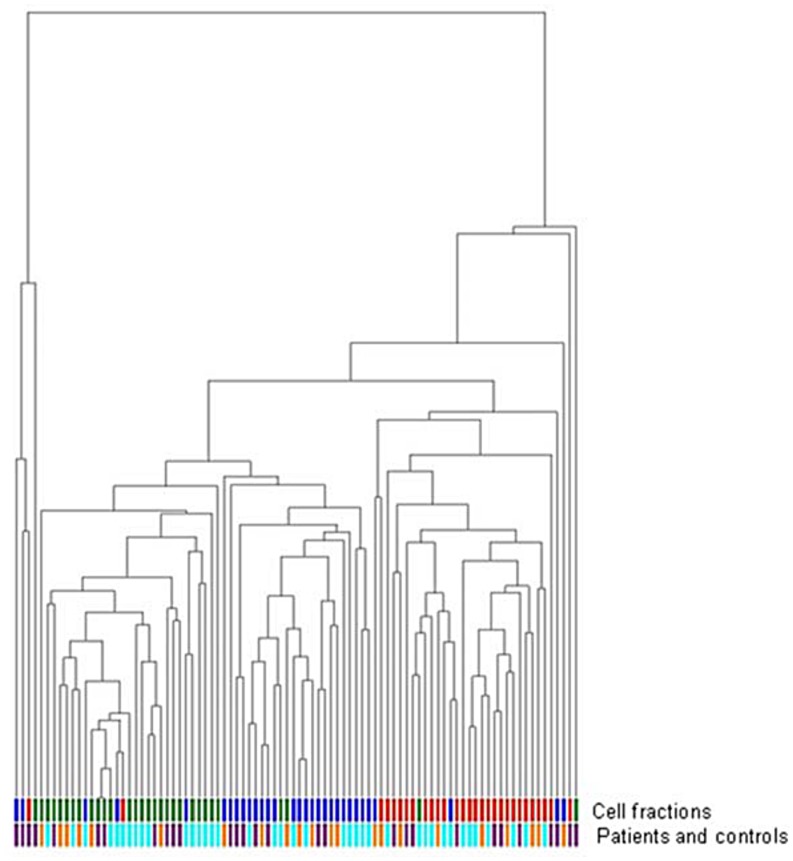
Unsupervised hierarchical cluster analysis of all samples based on apoptosis-related gene expression profile. Cell fractions are shown in the first column: CD34+ cells (blue), CD71+ cells (green), CD13/33+ cells (red). The second column shows patients and controls: controls (purple), low risk MDS (light blue), high risk MDS (orange).

Unsupervised hierarchical cluster analysis was performed based on the expression of 93 apoptosis-related genes. These included members of the TNF (Receptor) Family, BCL-2 Family, IAPs, Caspases, CARD Family, TRAF family and others (S2 Table). The analysis showed a clear clustering of the three hematopoietic cell fractions, indicating different patterns of apoptosis-related gene activity in these cell fractions (Fig 1). Unexpectedly, within each cell fraction, patients and healthy controls could not be distinguished based on the global expression pattern of these apoptosis-related genes (Fig 1). Also low-risk MDS and high-risk MDS patient groups could not be distinguished using this approach.

### Differential expression of apoptosis-related genes in MDS patients and controls

**Fig 2:**
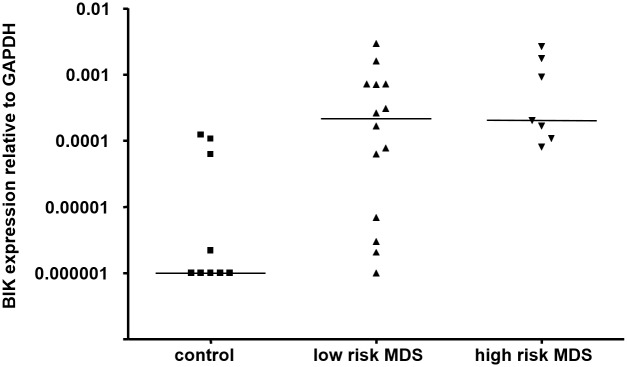
Expression of *BIK* in MDS patients and controls. *BIK* expression in ‘low risk’ patients, ‘high risk’ patients and controls. The expression of *BIK* is depicted relative to the housekeeping gene *GAPDH*. Median level of expression is indicated.

Analysis of individual genes revealed several genes that were differentially expressed between patients and controls in more than one cell fraction: *BIK*, *TNFRSF19* and *TNFRSF7* (CD34+ and CD71+ fractions) and *MDM2* (CD71+ and CD13/33+ fractions) (Tables 1, 2 and 3, Fig 2). Of these genes, only the pro-apoptotic *BIK* gene showed a significantly increased expression after correction of p-values for multiple testing. The analyses were repeated after the MDS patients were divided into low-risk and high-risk groups. As was the case for the comparison between all patients and controls, differential expression was detected of *TNFRSF4*, *TNFRSF19*, *RALBP1* and *TNFRSF7* in the CD34+ fraction, *BIK*, *DAPK1*, *DAPK2*, *BNIPL*, *TNFSRF10A*, and *CARD9* in the CD71+ group and *TNFSRF13B*, *MDM2*, *DAPK2*, *TNFRSF10C*, and *CARD6* in the CD13/33+ fraction. In addition, several other genes mainly showed differences in expression between the low-risk and high-risk MDS patients and not between patients and controls (S3 Table). *TNFRSF4* and *BIK* showed the most prominent differences in expression between the different patient groups and controls. *BIK* was expressed more than 200-fold higher in CD71+ cells of both low-risk MDS and high-risk MDS compared to healthy controls (p = 0.003). *TNFRSF4* displayed a marked down-regulation in CD34+ cells of low-risk MDS patients compared to controls, and a significant increase of expression in high-risk MDS CD71+ cells compared to low-risk MDS CD71+ (p = 0.0023).

**Table 1:** Differentially expressed genes in the CD34+ cell fraction in MDS patients versus controls.

Gene	p-value	effect on apoptosis	Ratio of median expression in patients/ controls
**TNFRSF19**	0.006	pro	0.2
**TNFRSF7**	0.013	pro	0.1
**BIK**	0.016	pro	0.6
**RIPK2**	0.020	pro	1.5
**CARD11**	0.035	pro	0.6
**RALBP1**	0.047	anti	2.9

**Table 2:** Differentially expressed genes in the CD71+ cell fraction in MDS patients versus controls.

Gene	p-value	effect on apoptosis	Ratio of median expression in patients/ controls
**BIK**	0.001*	pro	203
**BNIPL**	0.004	pro	0.1
**DAPK1**	0.020	pro	3.6
**BBC3**	0.015	pro	3.0
**EDA2R**	0.015	pro	49
**TNFRSF10A**	0.015	pro	3.9
**CARD9**	0.018	pro	1.6
**CASP9**	0.018	pro	7.0
**DAPK1**	0.012	pro	3.3
**MDM2**	0.020	anti	1.8
**TNFSRF19**	0.020	pro	0.2
**TNFRSF7**	0.022	pro	0.2
**TNFRSF10B**	0.028	pro	6.2
**BCL2L10**	0.032	anti	2.1
**BAG3**	0.035	anti	3.1
**TNFRSF10D**	0.044	anti	2.9

*indicates significant difference (p<0.05) using p-value for multiple testing

**Table 3:** Differentially expressed genes in the CD13/33+ cell fraction in MDS patients versus controls.

Gene	p-value	effect on apoptosis	Ratio of median expression in patients/ controls
**MDM2**	0.004	anti	1.7
**TNFRSF13B**	0.005	-	0.01
**FADD**	0.035	pro	1.7

## Discussion

We have studied the expression of a large set of genes and gene families that have been associated with apoptosis in three different FACS isolated hematopoietic cell fractions from MDS patients and healthy controls. Using sensitive real-time PCR analysis followed by unsupervised clustering analysis of the measured gene expression levels, we were able to distinguish the CD34+, CD71+ and CD13/33+ cell fractions. Unexpectedly, global patterns of gene expression could not distinguish healthy controls and low risk and high risk MDS patients, but consistently clustered the different cellular subsets. Gene expression analysis has been shown to be a powerful tool for patient stratification, discovery of cancer subtypes, and prediction of survival [33–40]. Although our cohort is relatively small, our results suggest that the apoptotic pathway is not grossly deregulated at the transcriptional level in MDS. Analysis of the expression of single genes identified a limited number of genes that were differentially expressed between these groups. Deregulation of the expression of some of these genes has been implicated in MDS before. Increased expression of *TNFRSF10A* (*TRAIL-R1*) in MDS has been described, though not specifically in the CD71+ population [23]. Differential expression of other genes, such as *TP53* and *BCL2*, that have previously been implicated in MDS, could not be confirmed in our study. Differences between our study and those of others could be due to the bone marrow fractions studied and the selection of patients. The *BIK* (BCL-2 interacting killer) gene showed the most prominent increased expression in CD71+ in MDS patients compared to controls. BIK is a pro-apoptotic BH3-only member of the Bcl-2 family [41]. Its binding to Bcl-2 promotes apoptosis [42] and such binding is elicited through several pathways, most notably TP53 [43], E2F1 [44], Smad [45], and miR608 [46]. *BIK* is normally undetectable or expressed at low levels in cancers [47–50] but specific tumors that were marked with high *BIK* expression have been targeted for induction of apoptosis leading to tumor reduction [51–54]. *BIK* has been found to be over-expressed in 14 out of 15 breast cancer samples [47], and was also present in a gene expression signature consisting of 64 genes that correlated with long-term survival in non-small cell lung cancer patients [55]. Although mutations in *BIK* have been found in B-cell lymphomas [56], little is known about the role of *BIK* in hematopoietic malignancies. In acute myeloid leukemia, *BIK* was found to be upregulated in the leukemic cells compared to controls [57]. In addition, it has recently been observed that BIK is involved in autophagy in cancer cells [58, 59]. Although the role of autophagy in myeloid malignancies is still largely unclear [60], autophagy inhibitors are currently being tested in clinical trials [61, 62]. The outstanding over-expression of *BIK* in MDS CD71+ cells is a novel finding, and since BIK is pro-apoptotic, its overexpression in MDS CD71+ cells might be involved in increased cell death, particularly in the CD71+ erythroid cells in MDS.

The only other gene that maintained significance after correction for multiple testing was *TNFRSF4*, which we found to be down-regulated in CD34+ cells of low risk MDS patients. TNFRSF4, also known as OX40 or CD134, is mainly known for its role in T-cells. Upon activation it promotes cell survival and clonal expansion [63], and the secretion of pro-inflammatory cytokines, mainly IL-2 [64]. TNFRSF4 has been suggested as a potential target for immunotherapy [65–67] [68]. Little is known of its direct involvement in tumorigenesis or cancer maintenance. It has been shown that the *TNFRSF4* gene is hypomethylated in MDS CD34+ cells [69] and a murine study has shown that expression of *TNFRSF4* increases upon progression from MDS to AML [70].

In conclusion, we show that unsupervised clustering of the gene expression level of a large set of apoptosis regulating genes does not identify gene signatures that are specific for MDS. Moreover, when single genes were regarded, only a limited number of apoptosis-regulating genes (BIK and TNFRSF4) were differentially expressed between normal and MDS derived cell fractions. This does however not exclude a role for (dysregulated) apoptosis in the pathogenesis of MDS, as we did not measure protein levels.

## Methods

### Patients and controls

We confirm that the study has been reviewed and approved by the Commissie Mensgebonden Onderzoek (CMO), the institutional review board (IRB) at the Radboudumc (NL), with the IRB file number CMO 2013/64 before the beginning of the study. For further details, contact the CMO at commissiemensgebondenonderzoek@radboudumc.nl or at the phone number 0031-24-3613154. Bone marrow from MDS patients (n = 23) and healthy controls (n = 10) was collected after informed consent. Written informed consent for the use of surplus diagnostic patient material was obtained in compliance with institutional regulations. Patients diagnosed at the Radboudumc belonging to all different WHO and IPSS categories were included. Patient characteristics are shown in S1 Table. Patient 11 had an exceedingly high granulocyte count. Nonetheless, the diagnosis of MDS RAEB1 was confirmed for this patient. All patient samples were collected before treatment was administered. Mononuclear cells were isolated from the bone marrow by density gradient centrifugation using Ficoll 1.077 g/mL (Pharmacia Biotech, Uppsala, Sweden) and viably frozen in liquid nitrogen until further use.

### Cell sorting and RNA isolation

After thawing, hematopoietic cell subtypes were isolated by fluorescence activated cell sorting (FACS) using monoclonal antibodies directed against CD45 and the side scatter profile. In addition, monoclonal antibodies directed against CD34, CD13, CD33 and CD71 were used. Gating on forward and side scatter was used to exclude dead cells and debris. RNA was isolated from three subfractions, the CD34+ stem/progenitor cells, the CD13/33+ mono/myeloid cells and the CD71+ erythroid cells.

### Gene expression analysis

In a first set of patients and controls, the expression of 180 different apoptosis-related genes was measured simultaneously by real-time PCR employing a 384 Microfluidic Card (Applied Biosystems). The genes measured and the corresponding assay numbers are shown in S2 Table. cDNA obtained from the CD34+ (patients n = 13, controls n = 5), CD71+ (patients n = 12, controls n = 4) and CD13/33+ (patients n = 11, controls n = 5) cell fractions was analyzed. Based on the expression pattern of these genes, a selection of 93 genes was used to perform a similar expression analysis in an additional group of CD34+ (patients n = 8, controls n = 5), CD71+ (patients n = 9, controls n = 5) and CD13/33+ cells (patients n = 8, controls n = 5). The relative expression of the apoptosis-related genes was calculated by measuring the housekeeping gene *GAPDH*. Relative expression values less than 1x10^-6^ were set to 1x10^-6^ to avoid bias introduced by including values reaching the limit of detection of the real-time PCR assay. In addition to the apoptosis-related genes, the expression of several genes implicated in erythroid differentiation (*TFRC*, *GYPA*, *GYPB*, *GYPC*, *SPTB*, *SPTA1*, *EBP41*, *EBP42* and *HBB*) was determined to check whether these genes indeed showed highest expression in the CD71+ cell fraction. The erythroid genes were only used as controls and not included in subsequent statistical analyses. Complete data are retrievable on Gene Expression Omnibus with the accession number GSE89579.

### Statistical analysis

Microfluidic card log-scale values were imported into Partek (Partek Genomic Suite software, version 6.5; Partek Inc., St. Louis, MO). Principle Component Analysis (PCA) showed a clear distinction between the three different tissues and thus the dataset was split accordingly into three separate groups. In each group expression differences between donors and patients was calculated using the non-parametric Mann-Whitney U-test. For statistical analysis, patients from the IPSS low and int-1 risk groups were combined in a ‘low risk MDS’ group and patients from the IPSS int-2 and high-risk groups in a ‘high-risk MDS’ group. Corresponding P-values were corrected for multiple testing by random permutations. The Kruskall-Wallis-test was used to identify differences between the ‘low risk MDS’, ‘high-risk MDS’ and control groups. In case of a p-value< 0.05, posthoc analysis was performed using non-parametric Mann-Whitney U test.

## Supporting Information

S1 Table Patients and controls. Patient characteristics: n/a = not available; * Normal Hemoglobin (Hb) range in adults = 2.0–2.7 mmol/L in males, 1.8–2.5 mmol/L in females; ** Normal granulocyte absolute count range in adults = 1.3–8.0 x10^9. Depending on material availability and quantity, it was possible to sort CD34+, CD71+, and CD13/33+ cell fractions from each patient.
(DOCX)

S2 Table Genes and assay numbers. Complete list of 384 apoptosis-related genes with Gene symbols, RefSeq record, assay number, and assay replicate
(DOCX)

S3 Table Differentially expressed genes. Genes with notable expression differences of patients versus low-risk MDS versus high-risk MDS in A) in the CD34+, B) in the CD71+, and C) in the CD13/33+ cell fraction. * indicates significant difference (p<0.05) in expression after posthoc analysis. ** indicates significant difference (p<0.05) using p-value for multiple testing.
(DOCX)
