# Supplementary material for: Apoptosis-Related Gene Expression Profiling in Hematopoietic Cell Fractions of MDS Patients
Source: PLoS One. 2016 Nov 30;11(11):e0165582. doi: 10.1371/journal.pone.0165582 (PMC5130187; doi:10.1371/journal.pone.0165582)
Supplement: S1 Table — Patient characteristics: n/a = not available; * Normal Hemoglobin (Hb) range in adults = 2.0–2.7 mmol/L in males, 1.8–2.5 mmol/L in females; ** Normal granulocyte absolute count range in adults = 1.3–8.0 x10^9. Depending on material availability and quantity, it was possible to sort CD34+, CD71+, and CD13/33+ cell fractions from each patient. (DOCX) [file pone.0165582.s001.docx]

| **No.** | **age** | **Diagnostic sample (sampling delay)** | | **FAB** | **WHO** | **IPSS** | **Karyotype** | **Blasts in BM** | **Hb (mmol/l)*** | **Granulocytes (x10^9/l)**** | **Cell fractions analyzed** |
| --- | --- | --- | --- | --- | --- | --- | --- | --- | --- | --- | --- |
| **patient 1** | n/a | | n/a | RA | RCMD | low | normal | 3 | 5.7 | 2.1 | CD34, CD13/33 |
| **patient 2** | 75 | | Yes | RA | RCMD | low | normal | 1 | 7.0 | 1.9 | CD34, CD71, CD13/33 |
| **patient 3** | 55 | | No (9 months) | RA | RCMD | low | normal | 1 | 3.8 | 5.8 | CD34, CD71, CD13/33 |
| **patient 4** | 63 | | No (8 months) | RARS | RCMD-RS | low | normal | 1 | 2.4 | 2.1 | CD34, CD71, CD13/33 |
| **patient 5** | 74 | | No (14 months) | RA | RA | int-1 | 46,XY, -20, +mar [10] | 3 | 6.2 | 2.6 | CD34, CD71, CD13/33 |
| **patient 6** | 52 | | Yes | RARS | RARS | int-1 | normal | 2 | 6.0 | 3.6 | CD34, CD71 |
| **patient 7** | 69 | | No (3 years) | RARS | RARS | int-1 | 47,XY,+8[4]/46,XY[6] | 2 | 5.6 | 2.4 | CD34, CD71, CD13/33 |
| **patient 8** | 52 | | No (3 months) | RARS | RCMD-RS | int-1 | normal | 1 | 3.4 | 0.7 | CD71 |
| **patient 9** | 67 | | No (6 years) | RAEB | RAEB-1 | int-1 | normal | 6 | 6.0 | 0.2 | CD34, CD71, CD13/33 |
| **patient 10** | 59 | | No (3 months) | RAEB | RAEB-1 | int-1 | normal | 9 | 7.5 | 4.5 | CD34, CD71, CD13/33 |
| **patient 11** | 59 | | No (5 months) | RAEB | RAEB-1 | int-1 | 47,XX,+8[7]/46,XX[3] | 6 | 6.5 | 28.1 | CD34, CD71, CD13/33 |
| **patient 12** | 66 | | No (3 months) | RAEB | RAEB-1 | int-1 | normal | 8 | 7.7 | 0.5 | CD34, CD71, CD13/33 |
| **patient 13** | 63 | | Yes | RAEB | RAEB-1 | int-1 | normal | 5 | 5.6 | 1.9 | CD34, CD71 |
| **patient 14** | 81 | | Yes | RAEB | RAEB-1 | int-1 | normal | 6 | 4.0 | 1.0 | CD34, CD71, CD13/33 |
| **patient 15** | 27 | | Yes | RAEB | RAEB-1 | int-1 | normal | 5 | 3.8 | 1.7 | CD71, CD13/33 |
| **patient 16** | 66 | | No (9 months) | RAEB | RAEB-2 | int-2 | normal | 4 | 6.6 | 2.5 | CD34, CD71, CD13/33 |
| **patient 17** | 53 | | Yes | RAEB-t | RAEB-2 | int-2 | normaal | 12 | 5.9 | 21.6 | CD34, CD71, CD13/33 |
| **patient 18** | 23 | | Yes | RAEB-t | RAEB-2 | int-2 | complex | 10 | 6.6 | 12.1 | CD34, CD71, CD13/33 |
| **patient 19** | 58 | | Yes | RAEB-t | AML | int-2 | normal | 21 | 6.7 | 2.5 | CD34, CD71, CD13/33 |
| **patient 20** | 61 | | Yes | RAEB-t | RAEB-2 | high | complex | 11 | 4.0 | 0.6 | CD34, CD71 |
| **patient 21** | 64 | | Yes | RAEB | RAEB-2 | high | 47,XY, +11 [5] / 46,XY [15] | 12 | 6.2 | 1.0 | CD34, CD13/33 |
| **patient 22** | 70 | | Yes | RAEB-t | AML | high | normal | 22 | 4.8 | 0.6 | CD34, CD71, CD13/33 |
| **patient 23** | 64 | | yes | RAEB-t | AML | high | complex | 22 | 6.3 | 1.3 | CD34, CD71, CD13/33 |
| **controls 1-9** | n/a | |  |  |  |  |  |  |  |  | CD34, CD71, CD13/33 |
| **control 10** | n/a | |  |  |  |  |  |  |  |  | CD34, CD13/33 |
