# Supplementary material for: Apoptosis-Related Gene Expression Profiling in Hematopoietic Cell Fractions of MDS Patients
Source: PLoS One. 2016 Nov 30;11(11):e0165582. doi: 10.1371/journal.pone.0165582 (PMC5130187; doi:10.1371/journal.pone.0165582)
Supplement: S2 Table — Complete list of 384 apoptosis-related genes with Gene symbols, RefSeq record, assay number, and assay replicate (DOCX) [file pone.0165582.s002.docx]

|  | | | |
| --- | --- | --- | --- |
|  |  |  |  |
| **Gene** | **NM number** | **Assay number** | **Assay** |
| **BAX** | NM_138761.2 | Hs00180269_m1 | 1,2 |
| **BBC3** | NM_014417.2 | Hs00248075_m1 | 1,2 |
| **BCL2A1** | NM_004049.2 | Hs00187845_m1 | 1,2 |
| **BCL2** | NM_000633.2 | Hs00153350_m1 | 1,2 |
| **BCL2L10** | NM_020396.2 | Hs00368095_m1 | 1,2 |
| **BCL2L11** | NM_138621.2 | Hs00197982_m1 | 1,2 |
| **BCL2L12** | NM_138639.1 | Hs00369327_m1 | 1,2 |
| **BCL2L13** | NM_015367 | Hs00209789_m1 | 1 |
| **BCL2L14** | NM_030766.1 | Hs00373302_m1 | 1,2 |
| **BCL2L1** | NM_001191 | Hs00169141_m1 | 1,2 |
| **BCL2L1** | NM_138578.1 | Hs00236329_m1 | 1,2 |
| **BCL2L2** | NM_004050 | Hs00187848_m1 | 1 |
| **BID** | NM_197966.1 | Hs00609630_m1 | 1,2 |
| **BIK** | NM_001197.3 | Hs00154189_m1 | 1,2 |
| **BLK** | NM_001715.2 | Hs00176441_m1 | 1,2 |
| **BNIP1** | NM_001205.1 | Hs00241824_m1 | 1,2 |
| **BNIP2** | NM_004330.1 | Hs00188939_m1 | 1,2 |
| **BNIPL** | NM_138278.2 | Hs00414503_m1 | 1,2 |
| **BOK** | NM_032515.3 | Hs00261296_m1 | 1,2 |
| **HRK** | NM_003806.1 | Hs00705213_s1 | 1,2 |
| **BAD** | NM_032989 | Hs00188930_m1 | 1 |
| **BAG1** | NM_004323 | Hs00185390_m1 | 1 |
| **BAG2** | NM_004282.2 | Hs00188716_m1 | 1,2 |
| **BAG3** | NM_004281.3 | Hs00188713_m1 | 1,2 |
| **BAG4** | NM_004874 | Hs00362193_m1 | 1 |
| **BAG5** | NM_004873 | Hs00191644_m1 | 1 |
| **BAK1** | NM_001188 | Hs00832876_g1 | 1 |
| **BCL10** | NM_003921 | Hs00184839_m1 | 1 |
| **BCL3** | NM_005178 | Hs00180403_m1 | 1 |
| **BCLAF1** | NM_014739 | Hs00602256_m1 | 1 |
| **BECN1** | NM_003766 | Hs00186838_m1 | 1 |
| **BMF** | NM_033503 | Hs00372938_m1 | 1 |
| **BNIP3L** | NM_004331 | Hs00188949_m1 | 1 |
| **C19orf6** | NM_033420 | Hs00364301_m1 | 1 |
| **MCL1** | NM_021960 | Hs00172036_m1 | 1 |
| **MCL1** | NM_182763 | Hs00766187_m1 | 1 |
| **PMAIP1** | NM_021127 | Hs00560402_m1 | 1 |
| **CARD10** | NM_014550.3 | Hs00367225_m1 | 1,2 |
| **CARD11** | NM_032415.2 | Hs00260906_m1 | 1,2 |
| **CARD12** | NM_021209.3 | Hs00368367_m1 | 1,2 |
| **CARD14** | NM_024110 | Hs00364499_m1 | 1 |
| **CARD15** | NM_022162.1 | Hs00223394_m1 | 1,2 |
| **CARD4** | NM_006092 | Hs00196075_m1 | 1 |
| **CARD6** | NM_032587.2 | Hs00261581_m1 | 1,2 |
| **CARD8** | NM_014959.1 | Hs00209095_m1 | 1,2 |
| **CARD9** | NM_052813.2 | Hs00364485_m1 | 1,2 |
| **NOL3** | NM_003946.3 | Hs00358724_g1 | 1,2 |
| **RIPK2** | NM_003821.5 | Hs00169419_m1 | 1,2 |
| **APAF1** | NM_181861 | Hs00559441_m1 | 1 |
| **C10orf97** | NM_024948 | Hs00227894_m1 | 1 |
| **C9orf89** | NM_032310 | Hs00260439_m1 | 1 |
| **COPl** | NM_052889 | Hs00430993_m1 | 1 |
| **NALP1** | NM_033004 | Hs00248187_m1 | 1 |
| **PYCARD** | NM_145183 | Hs00203118_m1 | 1 |
| **CASP3** | NM_032991.2 | Hs00234387_m1 | 1,2 |
| **CASP5** | NM_004347.1 | Hs00237061_m1 | 1,2 |
| **CASP6** | NM_032992.2 | Hs00154250_m1 | 1,2 |
| **CASP7** | NM_033338.4 | Hs00169152_m1 | 1,2 |
| **CASP8AP2** | NM_012115.2 | Hs00201640_m1 | 1,2 |
| **CASP8** | NM_033356.2 | Hs00154256_m1 | 1,2 |
| **CASP9** | NM_032996.1 | Hs00154260_m1 | 1,2 |
| **PACAP** | NM_016459.3 | Hs00414907_m1 | 1,2 |
| **CASP10** | NM_32974 | Hs00154268_m1 | 1 |
| **CASP14** | NM_012114 | Hs00201637_m1 | 1 |
| **CASP1** | NM_03329 | Hs00169146_m1 | 1 |
| **CASP2** | NM_032982 | Hs00154242_m1 | 1 |
| **CASP4** | NM_001225.3 | Hs00426677_m1 | 1 |
| **CASP8** | NM_033356.2 | Hs00236278_m1 | 1 |
| **CIDEC** | NM_022094.2 | Hs00535723_m1 | 1,2 |
| **CIDEA** | NM_198289 | Hs00154455_m1 | 1 |
| **CIDEB** | NM_014430 | Hs00205339_m1 | 1 |
| **CRADD** | NM_003805.3 | Hs00187009_m1 | 1,2 |
| **EDARADD** | NM_080738.2 | Hs00369830_m1 | 1,2 |
| **FADD** | NM_003824.2 | Hs00538709_m1 | 1,2 |
| **LRDD** | NM_018494 | Hs00388035_m1 | 1 |
| **MADD** | NM_130470 | Hs00366249_m1 | 1 |
| **TRADD** | NM_003789 | Hs00182558_m1 | 1 |
| **DEDD2** | NM_032998 | Hs00370206_m1 | 1 |
| **DEDD** | NM_133328 | Hs00172768_m1 | 1 |
| **BIRC3** | NM_182962.1 | Hs00154109_m1 | 1,2 |
| **BIRC7** | NM_139317.1 | Hs00223384_m1 | 1,2 |
| **BIRC8** | NM_033341.3 | Hs00364262_s1 | 1,2 |
| **BIRC1** | NM_004536 | Hs00244967_m1 | 1 |
| **BIRC2** | NM_001166 | Hs00357350_m1 | 1 |
| **BIRC4BP** | NM_017523 | Hs00213882_m1 | 1 |
| **BIRC4** | NM_001167 | Hs00236913_m1 | 1 |
| **BIRC5** | NM_001012271 | Hs00153353_m1 | 1 |
| **BIRC6** | NM_016252 | Hs00212288_m1 | 1 |
| **ABL2** | NM_005158 | Hs00246861_m1 | 1,2 |
| **ABL2** | NM_007314 | Hs00270858_m1 | 1,2 |
| **ATM** | NM_138292.3 | Hs00175892_m1 | 1,2 |
| **CHEK1** | NM_001274.2 | Hs00176236_m1 | 1,2 |
| **CHEK2** | NM_001005735.1 | Hs00200485_m1 | 1,2 |
| **DUSP2** | NM_004418.2 | Hs00358879_m1 | 1,2 |
| **GADD45A** | NM_001924.2 | Hs00169255_m1 | 1,2 |
| **MDM1** | NM_017440.2 | Hs00220015_m1 | 1,2 |
| **MDM2** | NM_002392.2 | Hs00242813_m1 | 1,2 |
| **PARK2** | NM_013987.1 | Hs00247755_m1 | 1,2 |
| **TP53BP1** | NM_005657.1 | Hs00152943_m1 | 1,2 |
| **TP53** | NM_000546.2 | Hs00153349_m1 | 1,2 |
| **TP73** | NM_005427.1 | Hs00232088_m1 | 1,2 |
| **TP73L** | NM_003722.3 | Hs00186613_m1 | 1,2 |
| **ABL1** | NM_005157 | Hs00245445_m1 | 1 |
| **AKT1** | NM_001014431 | Hs00178289_m1 | 1 |
| **GADD45B** | NM_015675 | Hs00169587_m1 | 1 |
| **P53AIP1** | NM_022112 | Hs00223141_m1 | 1 |
| **PARC** | NM_015089 | Hs00292746_m1 | 1 |
| **PERP** | NM_022121 | Hs00751717_s1 | 1 |
| **PIN1** | NM_006221 | Hs00749260_s1 | 1 |
| **PPM1D** | NM_003620 | Hs00186230_m1 | 1 |
| **PTEN** | NM_000314 | Hs00829813_s1 | 1 |
| **RCHY1** | NM_001008925 | Hs00295839_m1 | 1 |
| **RFWD2** | NM_022457 | Hs00375437_m1 | 1 |
| **RPA3** | NM_002947 | Hs00366098_m1 | 1 |
| **TP53BP2** | NM_005657.1 | Hs00610488_m1 | 1 |
| **PDCD1** | NM_005018.1 | Hs00169472_m1 | 1,2 |
| **PDCD4** | NM_145341.2 | Hs00377253_m1 | 1,2 |
| **PDCD5** | NM_004708.2 | Hs00270435_m1 | 1,2 |
| **FAS** | NM_152871.1 | Hs00531110_m1 | 1,2 |
| **TNFRSF10A** | NM_003844.2 | Hs00269491_m1 | 1,2 |
| **TNFRSF10B** | NM_147187.1 | Hs00366272_m1 | 1,2 |
| **TNFRSF10C** | NM_003841.2 | Hs00182570_m1 | 1,2 |
| **TNFRSF10D** | NM_003840.3 | Hs00174664_m1 | 1,2 |
| **TNFRSF11A** | NM_003839.2 | Hs00187189_m1 | 1,2 |
| **TNFRSF11B** | NM_002546.2 | Hs00171068_m1 | 1,2 |
| **TNFRSF13B** | NM_012452.2 | Hs00234859_m1 | 1,2 |
| **TNFRSF17** | NM_001192.2 | Hs00171292_m1 | 1,2 |
| **TNFRSF18** | NM_148901.1 | Hs00188346_m1 | 1,2 |
| **TNFRSF19** | NM_018647.2 | Hs00218634_m1 | 1,2 |
| **TNFRSF1B** | NM_001066.2 | Hs00153550_m1 | 1,2 |
| **TNFRSF25** | NM_148965.1 | Hs00237054_m1 | 1,2 |
| **TNFRSF4** | NM_003327.2 | Hs00533968_m1 | 1,2 |
| **TNFRSF7** | NM_001242.3 | Hs00154297_m1 | 1,2 |
| **TNFRSF8** | NM_152942.2 | Hs00174277_m1 | 1,2 |
| **TNFRSF9** | NM_001561.4 | Hs00155512_m1 | 1,2 |
| **CD40** | NM_152854 | Hs00374176_m1 | 1 |
| **FAF1** | NM_131917 | Hs00169544_m1 | 1 |
| **FBF1** | NM_001080542.1 | Hs00384673_m1 | 1 |
| **LTBR** | NM_002342 | Hs00158922_m1 | 1 |
| **TNFRSF12A** | NM_016639 | Hs00171993_m1 | 1 |
| **TNFRSF13C** | NM_052945 | Hs00606874_g1 | 1 |
| **TNFRSF14** | NM_003820 | Hs00187058_m1 | 1 |
| **TNFRSF19L** | NM_032871 | Hs00262701_m1 | 1 |
| **TNFRSF1A** | NM_001065 | Hs00533560_m1 | 1 |
| **TNFRSF21** | NM_014452 | Hs00205419_m1 | 1 |
| **EDA2R** | NM_021783.2 | Hs00222305_m1 | 1,2 |
| **TRAF1** | NM_005658.3 | Hs00194638_m1 | 1,2 |
| **TRAF6** | NM_145803.1 | Hs00377558_m1 | 1,2 |
| **TRAIP** | NM_005879.2 | Hs00183394_m1 | 1,2 |
| **TANK** | NM_133484 | Hs00370305_m1 | 1 |
| **TIFA** | NM_052864 | Hs00385268_m1 | 1 |
| **TRAF2** | NM_021138 | Hs00184186_m1 | 1 |
| **TRAF3** | NM_145726 | Hs00237035_m1 | 1 |
| **TRAF3IP2** | NM_147200 | Hs00210113_m1 | 1 |
| **TRAF4** | NM_145751 | Hs00188755_m1 | 1 |
| **TRAF5** | NM_145759 | Hs00182979_m1 | 1 |
| **TRAF7** | NM_032271 | Hs00260228_m1 | 1 |
| **TTRAP** | NM_016614 | Hs00213282_m1 | 1 |
| **AVEN** | NM_020371.2 | Hs00220565_m1 | 1,2 |
| **ENDOG** | NM_004435.2 | Hs00172770_m1 | 1,2 |
| **ICEBERG** | NM_021571.2 | Hs00253674_s1 | 1,2 |
| **AIF1** | NM_001623 | Hs00610419_g1 | 1 |
| **CYC1** | NM_001916 | Hs00357717_m1 | 1 |
| **DIABLO** | NM_138930 | Hs00219876_m1 | 1 |
| **HTRA2** | NM_013247 | Hs00234883_m1 | 1 |
| **DFFB** | NM_001004285.1 | Hs00237077_m1 | 1,2 |
| **DFFA** | NM_213566 | Hs00189336_m1 | 1 |
| **DAPK1** | NM_004938 | Hs00234480_m1 | 1,2 |
| **DAPK1** | NM_004938.2 | Hs00234489_m1 | 1,2 |
| **DAPK2** | NM_014326.3 | Hs00204888_m1 | 1,2 |
| **DAPK3** | NM_001348.1 | Hs00154676_m1 | 1,2 |
| **RALBP1** | NM_006788.3 | Hs00183639_m1 | 1,2 |
| **RIPK1** | NM_003804.3 | Hs00169407_m1 | 1,2 |
| **RIPK3** | NM_006871.3 | Hs00179132_m1 | 1,2 |
| **RIPK4** | NM_020639 | Hs00221005_m1 | 1 |
| **DAP** | NM_004394 | Hs00234397_m1 | 1 |
| **DAXX** | NM_001141969.1 | Hs00154692_m1 | 1 |
| **MIB1** | NM_020774 | Hs00379185_m1 | 1 |
| **BFAR** | NM_016561 | Hs00275423_m1 | 1 |
| **CFLAR** | NM_003879 | Hs00236002_m1 | 1 |
