# Supplementary material for: Apoptosis-Related Gene Expression Profiling in Hematopoietic Cell Fractions of MDS Patients
Source: PLoS One. 2016 Nov 30;11(11):e0165582. doi: 10.1371/journal.pone.0165582 (PMC5130187; doi:10.1371/journal.pone.0165582)
Supplement: S3 Table — Genes with notable expression differences of patients versus low-risk MDS versus high-risk MDS in A) in the CD34+, B) in the CD71+, and C) in the CD13/33+ cell fraction. * indicates significant difference (p<0.05) in expression after posthoc analysis. ** indicates significant difference (p<0.05) using p-value for multiple testing. (DOCX) [file pone.0165582.s003.docx]

| A) | | | | | |
| --- | --- | --- | --- | --- | --- |
|  |  |  |  |  |  |
|  |  |  | **Ratio of median expression in:** | | |
| **gene** | **p-value** | **effect on apoptosis** | **low risk MDS/ controls** | **high risk MDS/ controls** | **high risk MDS/ low risk MDS** |
| **TNFRSF4** | 0.0023** | anti | 0.2* | 0.9 | 4.4** |
| **TNFRSF19** | 0.0070 | pro | 0.3 | 0.1* | 0.2 |
| **RALBP1** | 0.031 | anti | 3.6* | 0.9 | 0.3 |
| **TNFRSF7** | 0.046 | pro | 0.2* | 0.1* | 0.4 |
| **TRAIP** | 0.050 | pro | 1.3 | 0.4 | 0.3* |
| **BNIP1** | 0.050 | anti | 1.9 | 0.8 | 0.4* |
|  |  |  |  |  |  |
|  |  |  |  |  |  |
| B) | | | | | |
|  |  |  | **Ratio of median expression in:** | | |
| **gene** | **p-value** | **effect on apoptosis** | **low risk MDS/ controls** | **high risk MDS/ controls** | **high risk MDS/ low risk MDS** |
| **BIK** | 0.003 | pro | 215* | 203* | 0.9 |
| **DAPK1** | 0.006 | pro | 2.5 | 6.8* | 2.8 |
| **DAPK2** | 0.008 | pro | 2.4 | 10.8* | 4.5* |
| **BNIPL** | 0.013 | pro | 0.1* | 0.1* | 0.5 |
| **TNFRSF10A** | 0.015 | pro | 3.2 | 8.1* | 2.5 |
| **CARD9** | 0.017 | pro | 1.3 | 3.3* | 2.6 |
| **BCL2L10** | 0.043 | anti | 1.1 | 3.4* | 3.0 |
|  |  |  |  |  |  |
|  |  |  |  |  |  |
| C) | | | | | |
|  |  |  | **Ratio of median expression in:** | | |
| **gene** | **p-value** | **effect on apoptosis** | **low risk MDS/ controls** | **high risk MDS/ controls** | **high risk MDS/ low risk MDS** |
| **TNFRSF13B** | 0.002 | - | 0.2* | 0.0* | 0.0* |
| **MDM2** | 0.011 | anti | 1.6* | 2.0* | 1.2 |
| **DAPK2** | 0.015 | pro | 0.5 | 1.9 | 3.9* |
| **TNFRSF10C** | 0.029 | anti | 0.5 | 1.7 | 3.5* |
| **CARD6** | 0.042 | anti | 0.9 | 2.3 | 2.5* |
